# Electrocardiographic Assessment of National-Level Triathletes: Sinus Bradycardia and Other Electrocardiographic Abnormalities

**DOI:** 10.3390/sports13010025

**Published:** 2025-01-16

**Authors:** Mike Climstein, Kenneth S. Graham, Michael Stapelberg, Joe Walsh, Mark DeBeliso, Kent Adams, Trish Sevene, Chad Harris

**Affiliations:** 1 School of Biological Science, Cumberland College of Health Sciences, Lidcombe, NSW 2141, Australia; 2 Department of Cardiology, Lidcombe Hospital, Lidcombe, NSW 2141, Australia; 3 Physical Activity, Lifestyle, Ageing and Wellbeing Faculty Research Group, University of Sydney, Sydney, NSW 2141, Australia; 4 Discipline of Exercise and Sport Science, Faculty of Medicine and Health, University of Sydney, Sydney, NSW 2141, Australia; 5 Specialist Suite, John Flynn Hospital, Tugan, QLD 4224, Australia; 6 AI Consulting Group, Sydney, NSW 2000, Australia; joe.walsh@scu.edu.au; 7 Sports Science Institute, Sydney, NSW 2000, Australia; 8 Faculty of Science and Engineering, Southern Cross University, Lismore, NSW 2480, Australia; 9 Ramp Physio and Fitness, Homebush, NSW 2140, Australia; 10 Department of Kinesiology and Outdoor Recreation, Southern Utah University, Cedar City, UT 84720, USA; 11 Kinesiology Department, California State University Monterey Bay, Seaside, CA 93955, USA; kadams@csumb.edu (K.A.);; 12 Exercise and Sport Sciences Department, Metropolitan State University of Denver, Denver, CO 80204, USA

**Keywords:** ECG, electrocardiography, athletes, endurance, bradycardic

## Abstract

Background: High-intensity endurance training induces specific cardiac adaptations, often observed through electrocardiographic (ECG) changes. This study investigated the prevalence of ECG abnormalities in national-level Australian triathletes compared to sedentary controls. Methods: A cross-sectional observational study was conducted involving 22 triathletes and 7 sedentary controls. Standard 12-lead ECGs assessed resting heart rate, ECG intervals, and axis deviation. Peak oxygen consumption was evaluated in triathletes to correlate with ECG indices and left ventricular mass, derived via echocardiography. Results: Triathletes exhibited significantly lower resting heart rates (53.8 vs. 72.1 bpm, −34%, *p* = 0.04), shorter QRS durations (0.088 vs. 0.107 ms, −21.6%, *p* = 0.01), and longer QT intervals (0.429 vs. 0.358 ms, +16.6%, *p* = 0.01) compared to controls. Sinus bradycardia was present in 68.2% of triathletes, with varying severity. First-degree atrioventricular block was identified in 13.6% of athletes, and left ventricular hypertrophy was confirmed in 18 triathletes via echocardiography. A significant positive relationship was identified between VO_2_peak and left ventricular mass (r = 0.68, *p* = 0.003). Conclusions: National-level triathletes exhibited ECG and structural cardiac adaptations consistent with high-intensity endurance training. Echocardiography is recommended for the accurate identification of LVH. These findings highlight the need for comprehensive cardiac evaluation in athletes to distinguish between physiological and pathological adaptations.

## 1. Introduction

The hearts of athletes have been of interest as early as the late 1800s when Henschen [1] conducted a physical examination using only percussion to distinguish the enlargement of the heart that he recognized was associated with athletic activity in cross-country skiers.

Modern cardiology and electrophysiology were founded during the late 19th and early 20th centuries when heart electrical conduction disorders were first reported in the scientific literature. Engelmann’s work in the late 1800s described anomalies in cardiac rhythm and set the foundation for understanding arrhythmias and conduction problems [2]. In the early 1900s, cardiac enlargement was identified with bradyarrhythmias in individuals with an increased exercise capacity [3]. Merghani et al. [4] stated that individuals who participated in a minimum of four hours of high-intensity exercise a week would induce myocardial adaptations (structural, functional, and electrical). Corrado and colleagues [5] are in agreement that myocardial remodeling, particularly the left ventricular posterior wall, interventricular septum, and ventricular and atrial enlargement, are commonly associated with electrical changes in the heart conduction system in athletes who train over long periods of time at a high intensity. These changes have also been observed in pediatric athletes [6,7].

High-intensity physical activity and exercise have been identified to be associated with conduction system abnormalities. One common electrocardiographic (ECG) abnormality is bradycardia (resting heart rate ≥ 60 to <100 bpm) [8], which has been anecdotally categorized as mild (resting HR of >50 to <59 bpm), moderate (resting HR of >40 to <49 bpm), and severe (resting HR of <40 bpm). The latter, severe bradycardia, is considered pathologic as opposed to physiologic, as a very low heart rate impairs the heart’s ability to pump oxygenated blood to the body’s organs and tissues. In all categories of bradycardia, there is typically a normal sinus rhythm where the pacemaking impulses originate in the sinoatrial node (SA node), progress to the atrioventricular junction (AV junction), and then to the ventricles via the His-Purkinje system [9]. Sinus bradycardia has been reported in retired athletes, highly trained athletes, and children. For example, Aagaard et al. [10] investigated conduction abnormalities in a cohort of 460 retired (mean age 55 years) National League American football players. They found the aged athletes had significantly lower heart rates at rest and a higher prevalence of first-degree atrioventricular block (1° AVB) compared to a non-elite athlete control group (18% vs. 9%, respectively). Abela and colleagues [11] also reported that a majority (80%) of highly trained athletes will demonstrate bradycardia, with a smaller percentage (7%) identified with 1° AVB. However, athletes with sinus bradycardia are generally asymptomatic, and it is considered a health adaptation [12].

Sheikh and Sharma [13] identified bradycardia in a 17-year-old athlete who was completing 20 h of swimming per week and cycling for 6 h per week. They reported that the athlete’s resting heart rate was 40 bpm. Bradycardia has also been reported in children. Doronia and Cherkasov [14] investigated resting heart rates in 83 children aged 8 to 12 who had two to three years of football training. They found that the majority (67%) of children had resting bradycardia.

Severe bradycardia (i.e., resting HR < 40 bpm) has been observed in a small percentage (4%) of highly trained endurance athletes [12]. Additionally, there have been reports of extreme bradycardia (i.e., resting HR < 30 bpm), mostly in endurance athletes [15]. Severe bradycardia has also been reported in an amateur soccer player, who had a resting heart rate during sleep of 33 bpm [16], and one athlete in her mid-50s with a resting heart rate of 29 bpm recorded while she was undergoing observation in an emergency department for an unrelated illness [17]. It should be noted that severe bradycardia during quiescent periods (i.e., rest or sleep) does not necessarily indicate a poor prognosis, and the majority of athletes with bradyarrhythmias are typically asymptomatic [3].

In addition to bradycardia, there are other ECG abnormalities reported in athletes. They include sinus arrhythmia, first-degree AV block, early repolarization, incomplete right bundle branch block, and left ventricular hypertrophy (LVH) by ECG voltage criteria. These electrocardiographic abnormalities can be seen in as many as 60% of athletes, either alone or in combination [18].

Corrado et al. [5] recommend classifying ECG abnormalities in athletes as those that are common versus those that are training-related. Training-related ECG changes commonly observed in athletes include sinus bradycardia, first-degree atrioventricular block, incomplete right bundle branch block, early repolarization, and QRS wave voltage criteria suggestive of LVH. 

Swimming, cycling, and running are all completed consecutively within the same event in a triathlon, which is recognized as a multidisciplinary sport. Training for three distinct sports necessitates scheduling numerous training sessions each day and week in addition to the high training volumes at high intensities required for endurance sports [19]. Training for triathletes requires them to complete aerobic and anaerobic exercise at high intensities. Aoyagi et al. [20] analyzed the exercise training intensities of 17 male TAs. They reported the mean percent of heart rate maximum of the TAs as 89% for swimming, 91% for cycling, and 90% for running. The aerobic capacity of triathletes has been reported in the literature; however, these studies primarily reported maximal aerobic power (i.e., VO_2_maxVO_2_peak) [21,22,23,24]. Although resting HR may be reported in these studies, electrocardiographic investigations of triathletes are limited and, to date, have only focused on ECG changes following a triathlon or triathlon training [25].

### Research Aim and Hypothesis

The research project aim was to comprehensively evaluate the prevalence and types of ECG abnormalities, including bradycardia, in national-level triathletes compared to sedentary controls and, additionally, to examine if resting blood pressure BP and other BP indices differ significantly between these two groups. There is scant literature that reports resting BP and ECG data on TAs. It was hypothesized the triathletes would exhibit a higher prevalence of resting bradycardia compared to sedentary controls. It was also hypothesized that other ECG abnormalities would be present, as well as lower resting heart rates, in TAs compared to sedentary controls.

## 2. Materials and Methods

### 2.1. Design and Participants

The Cumberland College of Health Sciences Human Research Ethics Committee provided approval for this cross-sectional observational study. The same 12-lead ECG and cardiologist were used to record and analyze the resting ECGs.

At the time of the study, a convenience sample of 22 triathletes (11 males and 11 females) who were members of Australia’s national triathlon team and attending a national training camp were informed of the study at the introduction session and invited to participate at that time. There were no exclusion criteria for any of the TAs attending the camp. However, their participation in the study was completely voluntary, and they could withdraw at any time with no penalty. The national training camp was invitation only, with TAs invited if they were “podium confirmation” (the TA had medaled at a pinnacle event or had been the World #1 at a world championship in the past 2 years) or “podium ready” (the TA displayed a performance capability trajectory capable of winning a medal at a pinnacle event). This aligns with the classification framework of Tier 3 (national level) to Tier 5 (World class) for athletes by McKay et al. [26].

Previous studies have shown that in order to alter myocardial mass, at least three hours of exercise per week are necessary [27]. Therefore, control participants were sedentary adults who were a convenience sample of medical doctors from various departments in the hospital. Control participants were deemed sedentary if they met the insufficient physical activity definition by the Australian Institute of Health and Welfare for adults 18 to 64 years of age (inclusive) who do not complete at least 150 min of moderate to vigorous activity across five or more days a week [28]. All doctors stated they had not participated in any form of regular physical activity or exercise for at least the previous five years, and they were not completing any physical activity during the study period. These sedentary controls stated their activity was limited to hospital rounds and incidental walking outside of work hours. Participants in the control group were required to be generally healthy nonsmokers, aged 16 to 35, who had no history of coronary heart or coronary artery disease, either past or present. Any potential controls who were older than 35 years or had a history of cardiovascular, metabolic, or neurological disease were ineligible to participate.

Physiological tests of peak oxygen consumption were all completed at the Cumberland College of Health Sciences, and all resting 12-lead ECGs and echocardiograms were completed at Ward 38, Cardiology at Lidcombe Hospital.

#### Research Aim and Hypothesis

There is scant literature that reports resting blood pressure and ECG data on TAs. Therefore, the research project aim was to comprehensively evaluate the prevalence and nature of ECG abnormalities, including bradycardia, in national-level triathletes compared to sedentary controls, and to examine if resting blood pressure and other parameters differ significantly between these two groups. It was hypothesized the triathletes would exhibit a higher prevalence of resting bradycardia compared to sedentary controls. It was also hypothesized that other ECG abnormalities would be present, as well as lower resting heart rates, compared to sedentary controls.

### 2.2. Measures

Following ethical approval from the Cumberland College of Health Sciences Human Research Ethics committee, all participants were informed of the testing procedures that would be undertaken and subsequently provided written informed consent. Data on athletes were collected over several days and included the determination of VO_2_peak (only TAs), a resting 12-lead electrocardiogram, and an echocardiographic/doppler examination.

#### 2.2.1. Anthropometric Measurements

Participants reported to the Cardiology ward at least two hours post-prandial and had not completed any heavy exercise for the previous 24 h. Standing height was measured to the closest 0.1 cm using a standard stadiometer (Seca GMBH, Hamburg, Germany). Participants’ body masses were determined with participants wearing only cycling shorts and thin T-shirts using a standard balance scale and measured to the nearest 100 grams (Seca GMBH, Hamburg, Germany).

#### 2.2.2. Blood Pressure

Following a 10 min quiescent period (seated, upright), resting systolic and diastolic blood pressure was assessed by a registered nurse via auscultation from the brachial artery using the standard clinical auscultation technique [29] with an aneroid sphygmomanometer (Gamma GT, Heine, Germany).

#### 2.2.3. Electrocardiographic Analysis

To collect the resting ECG, all participants were supine in the anatomical position on a hospital examination bed to minimize any movement artifacts. A clinical-grade electrocardiogram (Medtel (Old Greenwich, CT, USA), Cardiofax V model 8340) was used to attain standard 12-lead electrocardiograms (ECGs) from each participant prior to tests of peak oxygen consumption and, subsequently, an echocardiography examination. The ECG had inbuilt interpretation that provided analytical information/diagnosis pertaining to interval lengths, axis deviation [30], and other ECG abnormalities. Following each ECG recording, a hard copy 12-lead ECG was screened by the same registered cardiologist to ensure the participant was deemed safe to complete the maximal graded exercise tests (GXTs) (treadmill and cycle, triathletes only). The cardiologist then reviewed the ECG for conduction abnormalities and arrhythmias.

Heart rate was derived from the resting ECG. According to the manual, the ECG determined the heart rate by averaging six consecutive QRS complexes. Although the ECG updates the HR every two seconds, resting HR (bpm) was derived by checking two continuous QRS complexes via the hard copy, where 1500 was divided by the number of small boxes between two QRS complexes. The hard copy ECG also displayed the PR, QRS, and QT intervals (msec).

#### 2.2.4. Maximal Oxygen Consumption Testing

The gold standard for determining endurance capacity is widely acknowledged in the literature to be maximal, symptom-limited exercise testing [31]. Therefore, symptom limited, graded exercise tests were conducted on the TAs to assess their endurance capacity. The triathletes’ physiological testing included peak oxygen consumption on both a treadmill and an electronic cycle ergometer. The custom-designed, open-circuit spirometry system consisted of an oxygen analyzer (Ametek (Berwyn, PA, USA): Applied Electro Chemistry, model MN-22m), carbon dioxide analyzer (Ametek: Applied Electro Chemistry, model P-61B), flow control meter (Ametek: Applied Electro Chemistry, model R2), and Hewlett Packard flow transducer/pneumotach (47304A). These devices were all interfaced with a PC computer to provide real-time, online measurements of metabolic (VO_2_ L/min, VO_2_ mL/kg/min) and respiratory (VE) outcome measures. To ensure the accuracy of the open-circuit spirometry system, the flow transducer was calibrated prior to each VO_2_ test using a Tissot spirometer. Gas analyzers were also calibrated prior to and immediately after each VO_2_ test using certified reference gases (Commonwealth Industrial Gases). Maximal metabolic and respiratory outcome variables were measured directly and analyzed every 30 s via the open-circuit spirometry technique [32]. Metabolic values were corrected via regression where O_2_ and/or CO_2_ analyzers demonstrated drift. The expired volume was calibrated by using a 3.0-L Vac-U-Med calibration syringe. All analyzers were sampled at 100 Hertz, and STPD values for VE (L/min), VO_2_ (L/min), and VCO_2_ (L/min) were calculated and displayed, along with heart rate at 30 s intervals.

Using a typical ramping protocol, the TAs were randomly assigned to undertake continuous, incremental GXTs on a customized wide-body treadmill and a cycle ergometer (Siemens 380B electrically braked cycle ergometer, Siemans Elema, Solna, Sweden) in order to measure their VO_2_ peak. Control participants did not complete any VO_2_ peak testing.

For both testing modes, a continuous, incremental approach was employed. After a two-minute warm-up, the gender-specific cycle GXT protocol used 20 watts for the first minute with 20 watt increments per minute for females and 30 watts for the first minute with 30 watt increments per minute for males. Participants self-selected their pace for the treadmill testing, which ranged from 10 to 14 km per hour.

A continuous, incremental protocol was used for both modes of testing. The cycle GXT protocol, gender-specific, utilized a 2 min warm-up, then 20 watts for the first minute and 20 watt increments per minute (females) and 30 watts for the first minute and 30 watt increments per minute (males). For treadmill testing, participants ran at a self-selected pace ranging from 10 to 14 km per hour. Following a 0% grade warm-up, the treadmill grade was raised by 2.0% each minute. GXTs were performed on a treadmill and a bicycle until voluntary fatigue. We averaged the four peak values that the Exerstress metabolic system (Sydney, Australia) recorded during the final 2 min of exercise. The maximum oxygen consumption was determined with the VO_2_ peak calculated as the average of the top two consecutive readings [33]. VO_2_ peak tests were scheduled at least 48 h apart in an effort to minimize any fatigue on the TAs.

### 2.3. Statistical Analysis

Statistical significance between groups was determined with a significance level set a priori at *p* < 0.05. Results were presented as means and standard deviations (±SDs). Initially, kurtosis, skewness, and QQ plots were examined (both visually and analytically) to determine data normality. Additionally, we applied a Lilliefors significance correction to the Kolmogorov–Smirnov test. Levene’s test for equality of variances was used to evaluate heteroscedasticity. To determine significant differences between TAs and sedentary controls, a one-tailed independent samples t-test was employed. This statistical test assessed variations in key variables such as resting HR, intervals (PR, QRS, QT), and axis deviation. The choice of the one-tailed test aligns with the hypothesis-driven approach to detect specific directional differences related to cardiac adaptations in athletes completing high-intensity training [5,18]. A one-way analysis of variance (ANOVA) with a Tukey’s post hoc test was used to analyze differences between subgroups (male and female TAs and controls).

All statistical analyses were completed using SPSS (version 27.0), ensuring a robust platform for data processing. Additionally, correlations between variables such as VO_2_ peak and left ventricular mass were evaluated using Pearson’s correlation coefficient to explore relationships between physiological adaptations and aerobic capacity. These methods provided a comprehensive evaluation of the cardiac profiles of the study participants, enabling the identification of significant electrocardiographic differences and their implications in the context of high-intensity endurance training.

## 3. Results

A total of 22 triathletes and 7 sedentary controls participated in this study with no adverse outcomes reported during any of the tests. The training and competition histories of the TAs are depicted in Table 1. TAs reported longer lengths of training and competition in swimming, followed by running and cycling.

All TAs completed peak oxygen consumption testing on both an upright electronically braked cycle ergometer and a motorized treadmill, in a randomized order. We found the VO_2_ peak for TAs was significantly higher (*p* = 0.015) for the treadmill test compared to the cycle ergometer test (+9.5%) (Table 2). There was a significant (*p* = 0.001) positive correlation between the treadmill and cycle tests (r = 0.909).

We identified no significant differences in participant characteristics (Table 3); however, TAs were slightly younger in age (−11.4%), negligibly taller in stature (+0.01%), and slightly heavier in mass (+2.0%) compared to controls. Additionally, there were no differences between groups with body mass index (BMI) or body surface area (BSA), with TAs slightly greater than controls (+3.7% and +2.3%, respectively). There were no significant differences between groups in resting SBP or DBP; however, TAs had slightly lower readings (−0.1% and −8.6%, respectively). Further assessment of resting BP indices revealed no differences in pulse pressure (PP) or mean arterial pressure (MAP) between groups.

**Figure 1:**
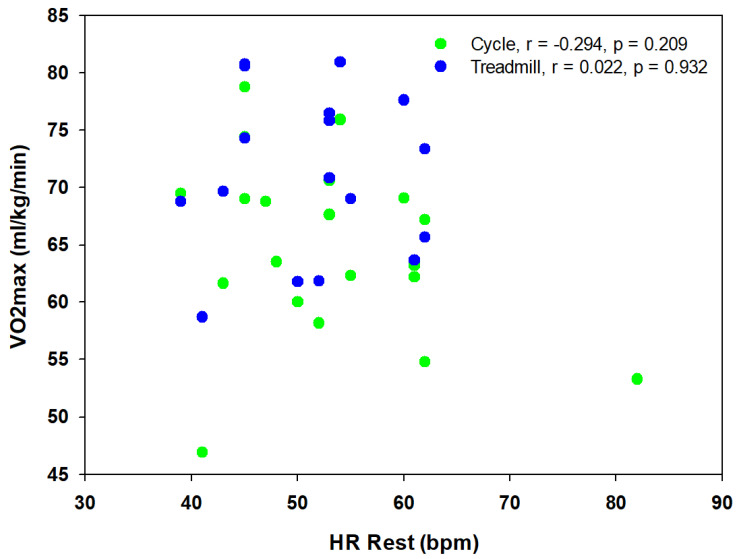
Relationship between resting heart rate (HR Rest) and VO_2_ peak for cycle and treadmill tests.

**Figure 2:**
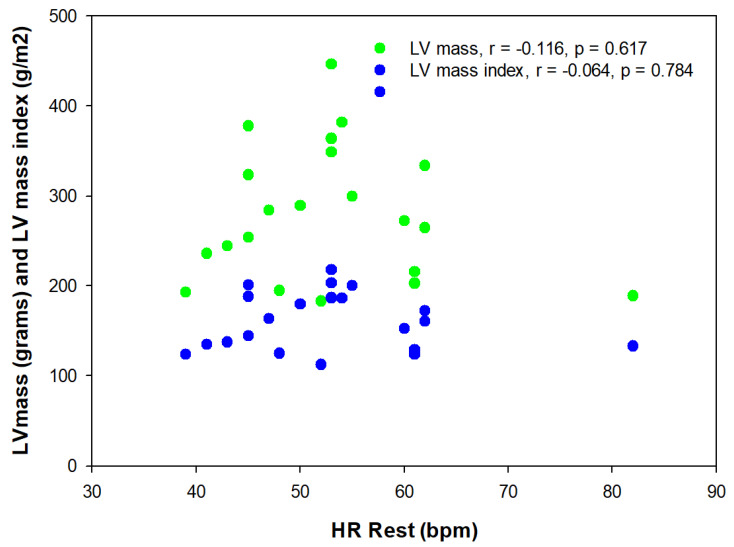
Relationship between resting heart rate (HR Rest) and left ventricular mass.

There was a difference in resting HR, with TAs having a significantly lower (*p* = 0.04) HR at rest compared to sedentary controls (−34.0%). There was no correlation between resting HR and VO_2_ peak (cycle or treadmill, Figure 1) or left ventricular mass (absolute or relative, Figure 2).

We did not identify a difference in the PR interval between the groups. However, TAs had a slightly longer PR interval (+7.2%). We identified that TAs had a significantly shorter QRS interval (*p* = 0.01, −21.6%), a longer QT interval (*p* = 0.01, +16.6%), and an axis deviation, albeit normal, significantly more oriented towards left axis deviation (*p* = 0.05, 45% [relative]) (Table 4).

There are notable gender differences in ECG intervals among athletes reported in the literature, and these differences are influenced by physiological, hormonal, and structural variations between male and female athletes. We, therefore, further analyzed the ECG data with TA gender as a subset. However, we found no significant differences between TA genders with regard to PR interval (*p* = 0.30), QRS interval (*p* = 0.276), or QT interval (*p* = 0.705). We did, however, identify a significant difference in the QRS interval between female TAs and controls, with the female TAs having a shorter QRS interval (0.083 vs. 0.107 msec, *p* = 0.001). We also identified significant differences (*p* < 0.001) in the QT interval between both male and female TAs compared to controls (0.421, 0.431 vs. 0.358 msec, respectively).

With regard to ECG abnormalities, there were no conduction abnormalities or arrhythmias noted in the control group. The majority (68.2%), however, not all, of the TAs demonstrated sinus bradycardia. A total of seven TAs demonstrated mild bradycardia (HR rest ≥ 50 to ≤59 bpm), eight TAs demonstrated moderate bradycardia (HR rest ≥ 40 to ≤49 bpm), and one TA demonstrated severe bradycardia (<40 bpm).

Concerning conduction anomalies, three TAs were found to have first-degree AV block (1° AVB), one TA was found to have a prolonged QRS interval, one TA was found to have a prolonged Qt interval (however, not long-QT syndrome), three TAs were identified with sinus arrhythmia, two TAs had tall T waves (chest leads), and four TAs had ECG voltage criteria that was indicative of left ventricular hypertrophy (LVH) (S wave depth in V1 plus the tallest R wave height in V5 or V6 is greater than 35 mm).

We had two TAs who exhibited multiple abnormalities on their ECGs. One TA had 1° AVB and voltage criteria indicative of LVH. The other TA had tall T waves and voltage criteria indicative of LVH.

Given that LVH was identified via ECG in a number of TAs, we also investigated LVH via echocardiography using the Penn equation (LV mass = 1.04 [(LVD + IVS + PW)^3^ − LVD^3^] − 13.6 g) [34]. We found that TAs had a significantly higher estimated LVmass (*p* = 0.001) compared to sedentary controls (283.8 vs. 130.1 g, +54.1%). We also identified a significant (*p* = 0.003) positive correlation (r = 0.680) between treadmill VO_2_ peak and left ventricular mass and cycle VO_2_ peak and left ventricular mass (*p* = 0.003, r = 0.608).

We identified a significantly higher (*p* = 0.002) LVmass index in triathletes versus controls (161.7 versus 79.2 g/m^2^, +104%). Although four TAs were identified with LVH via electrocardiography, echocardiography actually identified eight TAs with an absolute left ventricular mass > 300 g, indicative of LVH [35], and when considered relative to body mass (>3.5 g/kg), we identified 18 TAs who met this criteria [36]. No controls were identified via echocardiography as having LVH. Electrocardiographic criteria to estimate LVH have been well investigated in the literature. When analyzing TA subgroups and controls for LVmass and LVmass index, we identified significant differences (*p* < 0.001) between TAs (males vs. females) and both genders versus controls (*p* < 0.001). Male TAs’ LVmass was 32.5% greater than that of female TAs, and the males’ LVmass index was 22.1% greater than that of female TAs. With regard to LVmass, male and female TAs had significantly (*p* < 0.001) higher mass than controls (339.44, 228.26, and 130.11 g, respectively) and a significantly (*p* < 0.001) higher LVmass index (181.77, 141.68, and 81.91 g/m^2^, respectively).

## 4. Discussion

The purpose of this study was to determine if electrocardiographic abnormalities, including bradycardia, existed in national-level triathletes. Our main finding was national-level triathletes were not immune from electrocardiographic abnormalities; a high percentage were identified with bradycardia; however, not all TAs, despite very high aerobic fitness, were identified with bradycardia.

Surprisingly, we did not identify any differences between groups with regard to the hemodynamics of resting blood pressure and blood pressure-related indices of PP and MAP at rest. There was a non-significant difference of −8.6 percent in TAs with regard to DBP; we believe this lack of significance between groups may be attributed to the low number of participants in both groups. The lack of significant differences in blood pressure-related indices, such as PP and MAP, suggest that high-intensity training did not adversely affect hemodynamic stability at rest.

There is scant literature that reports resting blood pressure in TAs; however, our TAs’ values are similar to those reported by Leischik et al. [37], who reported outcome variables, including SBP, by the amount of LVmass in their participants. For TAs in that study with an LVmass < 220 g, the mean SBP was 125.5 mmHg, and for the TAs with an LVmass > 220 g, SBP was 130.8 mmHg. The mean resting SBP by Leischik et al. [37] was higher (+10.6%) than we found in the TAs in our study. The mean TAs in our study would be classified as normotensive, whereas the participants in Leischik et al. [37] would be classified as elevated, according to the American College of Cardiology [38]. Unfortunately, they did not report resting diastolic blood pressure. Douglas et al. [39] assessed resting BP in 235 TAs who were participating in the Hawaiian Ironman Triathlon and reported a similar mean elevated value of 122/74 mmHg. Sharwood and colleagues [40] reported the mean resting SBP and DBP in South African TAs, which were also higher than we found in our study (SBP 133 mmHg/DBP 78 mmHg).

The peak oxygen consumption measured in our TAs was similar to values previously reported in the literature for treadmill running (63.2 to 78.5 mL/kg/min) and cycling (61.3 to 75.9 mL/kg/min) [41,42]. Given our TA training and competition experience and peak oxygen consumption, we believe these metrics support the elite status of our participants and should have been an adequate stimulus to induce bradycardia. However, only 68.2% of the TAs demonstrated resting bradycardia to varying degrees. The bradycardia seen in endurance athletes is a hallmark of the athlete’s heart, a physiologic adaptation. As not all TAs demonstrated bradycardia, despite their high aerobic fitness, this is suggestive of individual variability in cardiac conduction adaptations. Bahrainy and colleagues [43] investigated bradycardia using parasympathetic withdrawal using isoproterenol and concluded that bradycardia was most likely due to a decrease in intrinsic heart rate.

Bradycardia is not uncommon in endurance athletes, with 50–85% of all athletes and more than 90% of endurance athletes reported to have resting bradycardia [44]. Although a lower percentage of our TA participants were found to have bradycardia, this is not an unrealistic finding. When we correlated resting HR with age, we found a non-significant correlation (*p* = 0.694, r = −0.091).

The PR interval represents the time between the onset of atrial depolarization and ventricular depolarization, and it has been reported in the literature that the PR interval tends to be shorter in female athletes compared to their male counterparts; however, this was not observed in this study. This difference is partly due to variations in autonomic tone, with females generally exhibiting higher vagal tone and heart rates, which can shorten conduction intervals [45].

Other ECG findings included a significantly shorter QRS interval in TAs (−21.6%, *p* = 0.01) and a longer QT interval (+16.6%, *p* = 0.01) compared to controls. These findings may reflect enhanced ventricular conduction and repolarization changes associated with myocardial conduction adaptations to endurance training [5]. The QRS duration reflects ventricular depolarization and is generally slightly shorter in female athletes than in males, which is what we observed in this study; however, there were no significant differences between TA genders (*p* = 0.08). The lengthened QRS interval seen in males is likely influenced by differences in ventricular mass, as males tend to have larger left ventricular mass, leading to longer depolarization times [46].

The QT interval represents the total duration of ventricular depolarization and repolarization and is consistently longer in female athletes, which we observed in this study, albeit non-significantly (*p* = 0.456). This gender difference is attributed to the influence of sex hormones, particularly estrogen, which has been shown to affect ventricular repolarization [47]. This can have clinical implications, as prolonged QTc intervals are associated with a higher risk of arrhythmias in females.

Additionally, TAs exhibited a significant (*p* = 0.05) leftward axis deviation, consistent with left ventricular remodeling seen in athletes. LVH in athletes results from the increased hemodynamic load imposed by regular, high-intensity training. Endurance training predominantly leads to eccentric hypertrophy, which is characterized by an increased cavity size with proportional wall thickness. The leftward axis deviation shift we observed in our TAs reflects the dominance of the hypertrophied left ventricle.

There are clinical ramifications for an athlete’s axis deviation towards LVH, as the risk of arrhythmias increases with increases in myocardial mass. The risk is believed to be the prolonged myocardial conduction pathways that predispose athletes to arrhythmias.

Only a small percent (13.6%) of our TAs were found to have 1° AVB, which is higher than that previously reported by Drezner et al. [48] in their consensus statement of electrocardiography in athletes of approximately 7.5%. Although 1° AVB is generally asymptomatic, it may indicate increased vagal tone or structural changes in the atrioventricular node. Additionally, 1° AVB has been reported to lead to a greater risk of arrhythmias in the future [49].

Electrocardiography identified 4 TAs with LVH, whereas follow-up testing with echocardiography revealed 8 TAs with LVH (via absolute criteria) and 18 TAs with LVH (via relative criteria). This underscores the limitations of ECG in detecting LVH, as only 4 athletes were identified with LVH by ECG criteria compared to 18 by echocardiography. For LVH detection, echocardiography has been shown to be more sensitive than ECG. Therefore, it is not surprising that additional TAs were identified with LVH via the echocardiogram investigation.

The strong positive correlation between VO_2_ peak and left ventricular mass (r = 0.68, *p* = 0.003) suggests that aerobic capacity directly influences myocardial remodeling. This highlights the importance of echocardiographic assessment in differentiating physiological from pathological adaptations in athletes.

### Strengths and Limitations

This study showcases several key strengths that underscore its scientific contribution. Firstly, the study design effectively contrasts a cohort of elite Australian triathletes with age-matched sedentary controls. This approach highlights the physiological and electrocardiographic adaptations resulting from high-intensity endurance training, such as sinus bradycardia, LVH, and conduction anomalies. The inclusion of both electrocardiographic and echocardiographic evaluations enriches the study by not only identifying abnormalities, but also providing detailed insights into the structural and functional cardiac changes that occurred in the TAs, particularly the limitations of ECG for diagnosing LVH compared to echocardiography. Significant ECG differences detected may have implications for future understanding and optimizing programming design for triathlon and potentially other endurance athletes.

Another significant strength is the rigorous methodology, including standardized data collection protocols and robust statistical analyses. The detailed characterization of participants, combined with precise diagnostic tools, ensures the reliability of the findings. The study’s focus on elite TAs, an under-researched athletic population, provides valuable data that contributes to the broader understanding of “athlete’s heart” phenomena. By addressing both common and training-related ECG changes, this research offers practical insights for clinicians involved in pre-participation screening and the management of athletes, emphasizing the importance of distinguishing physiological adaptations from pathological conditions.

This study has several limitations to be considered when interpreting the findings. The small sample size of 22 triathletes and 7 sedentary controls restricts the generalizability of our results to broader populations. Additionally, the reliance upon a single resting 12-lead electrocardiogram (ECG) may have limited the detection of transient or exercise-induced abnormalities, as extended monitoring, such as 24 h Holter monitoring, was not performed. The control group, comprised of sedentary medical professionals, may not fully represent the general sedentary population due to potential differences in occupational stress and baseline health metrics. Furthermore, the cross-sectional design of the study precludes causal inferences and longitudinal analyses of changes in the cardiac profiles of athletes over time. Additionally, although we reported the training and competition history of the TAs, we did not survey or have access to the TAs’ training regimes such as training intensity, training session durations, and percentage of aerobic versus anaerobic training. We also did not inquire into any injuries or illness that would have impaired the TAs’ training progress.

Moreover, the study’s scope was limited to echocardiographic and electrocardiographic measurements, without accounting for other potentially influential factors such as genetic predispositions, diet, and comprehensive exercise history. Additionally, due to time constraints by participants and cardiologists, we were unable to re-test participants or have a second cardiologist complete echocardiographic measurements. Recruitment from a national training camp also introduces potential selection bias, as it may have included only the most elite triathletes, excluding other subgroups within the athletic population. These limitations underscore the need for further research with larger, more diverse cohorts and a more comprehensive range of assessments to validate and expand upon these findings.

## 5. Conclusions

This study highlights the significant myocardial conduction adaptations associated with high-intensity training in elite Australian triathletes. The observed prevalence of sinus bradycardia and other electrocardiographic abnormalities, including LVH and conduction anomalies, underscores the profound physiological remodeling that occurs in response to sustained, high-intensity endurance exercise. Notably, while 68.2% of triathletes exhibited some degree of bradycardia, the variability in ECG findings suggests individual differences in cardiac adaptation, even among athletes of similar training intensity and aerobic capacity. Furthermore, the limitations of ECG in diagnosing LVH, compared to echocardiography, reinforce the need for comprehensive cardiac evaluation in athletic populations if the diagnosis of LVH is a key outcome variable.

These findings contribute to the growing body of literature on the “athlete’s heart”, providing insights into the cardiac profiles of elite triathletes and emphasizing the importance of distinguishing physiological adaptations from pathological conditions. Future research with larger and more diverse cohorts is necessary to explore the long-term implications of these adaptations and refine screening criteria for cardiovascular abnormalities in athletes.

## Figures and Tables

**Table 1:** Triathletes’ training and competition experience, where values are displayed as mean ± SD.

Event	Training (yrs) (Mean)	Training (yrs) (±SD)	Competition (yrs) (Mean)	Competition (yrs) (±SD)
Swimming	5.8	4.1	5.5	3.6
Cycling	3.1	2.1	2.9	1.9
Running	4.5	4.2	3.8	3.0

**Table 2:** Triathletes’ peak oxygen consumption on cycle and treadmill, where values are displayed as mean ± SD.

Mode	Mean	(±SD)
Cycle (mL/kg/min)	64.9	7.7
Treadmill (mL/kg/min)	71.1	7.1

**Table 3:** Participant physical and hemodynamic characteristics. Values are displayed as mean ± SD.

	Triathletes (*n* = 22)		Controls (*n* = 7)		*p*-Value
	Mean	±SD	Mean	±SD	
Age (years)	20.6	4.1	23.3	4.3	NS
Height (m)	169.1	9.8	168.8	8.0	NS
Mass (kg)	62.9	7.7	61.7	8.5	NS
BMI (kg/m^2^)	21.9	1.3	21.1	1.8	NS
BSA (m^2^)	1.74	0.17	1.70	0.19	NS
Rest SBP (mmHg)	112.2	11.2	113.0	11.9	NS
Rest DBP (mmHg)	67.2	7.1	73.0	7.9	NS
Pulse Pressure (mmHg)	45.0	10.4	40.0	5.0	NS
MAP (mmHg)	82.1	7.2	86.2	9.2	NS

*Where***:** SD = standard deviation, m = meters, kg = kilograms, BMI = body mass index, BSA = body surface area, SBP = systolic blood pressure, DBP = diastolic blood pressure, MAP = mean arterial pressure.

**Table 4:** Electrocardiographic results, where values are displayed as mean ±SD.

ECG Variable	Triathletes (Mean)	Triathletes (±SD)	Controls (Mean)	Controls (±SD)	*p*-Value
HR rest (bpm	53.8	10.3	72.1	8.1	0.04
PR interval (ms)	0.167	0.04	0.155	0.02	NS
QRS interval (ms)	0.088	0.01	0.107	0.01	0.01
QT interval (ms)	0.429	0.03	0.358	0.02	0.01
Axis deviation (degrees)	38.7	21.2	56.1	19.3	0.05

## Data Availability

The data presented in this study are available on request from the corresponding author due to ethical restrictions.

## Abbreviations

The following abbreviations are used in this manuscript:

ECG	Electrocardiogram
LVH	Left ventricular hypertrophy
HR	Heart rate
BPM	Beats per minute
AV	Atrioventricular
AVB	Atrioventricular block
VO_2_	Peak oxygen consumption
TA	Triathlete
GXT	Graded exercise test
VE	Ventilation
O_2_	Oxygen
CO_2_	Carbon dioxide
STPD	Standard temperature pressure dry
L	Liters
Min	Minute
SD	Standard deviation
M	Meters
Kg	Kilograms
BMI	Body mass index
BSA	Body surface area
SBP	Systolic blood pressure
DBP	Diastolic blood pressure
MAP	Mean arterial pressure
1° AVB	First-degree atrioventricular block
